# Insights into the Cenozoic geology of North Beirut (harbour area): biostratigraphy, sedimentology and structural history

**DOI:** 10.14324/111.444/ucloe.000004

**Published:** 2020-02-10

**Authors:** Germaine Noujaim Clark, Marcelle BouDagher-Fadel

**Affiliations:** 1 Independent Consultant geologist and Research associate to Lebanon National Council for Scientific Research, Lebanon; 2 Office of the Vice-Provost (Research), University College London, 2 Taviton Street, London WC1H 0BT, UK

**Keywords:** Foraminifera, Cenozoic, biostratigraphy, sedimentology, palaeoenvironment, paleogeography, regional tectonics

## Abstract

The biostratigraphy and sedimentology of the outcrops and bedrock recently exposed in archaeological excavations around the harbour area of Beirut (~5 km²) unlock the geological and structural history of that area, which in turn are key to understanding the hydrocarbon and hydrogeological potential of the region. A key location (Site 2) of a studied outcrop section and newly uncovered bedrock is on the northern foothill cliff of East Beirut (Achrafieh). The outcrop section of carbonates is of Eocene beds overlain by conformable Miocene beds. The excavation of the slope bordering the outcrop uncovered a bedrock section of an early Pliocene shoreline of carbonate/siliciclastic sands at its base and topped by a beach-rock structure. The early Pliocene age of the shoreline section is dated by an assemblage of planktonic foraminifera that includes *Sphaeroidinellopsis subdehiscens*, *Sphaeroidinella dehiscens* and *Orbulina universa*. The Eocene carbonates of Site 2 extend the coverage of the previously reported Eocene outcrops in the harbour area. They form a parasequence of thin-bedded, chalky white limestones that includes the youngest fossil fish deposits in Lebanon (*Bregmaceros filamentosus*). The deposits are dated as early Priabonian by their association with the planktonic foraminiferal assemblage of *Porticulasphaera tropicalis*, *Globigerinatheka barri*, *Dentoglobigerina venezuelana*, *Globigerina praebulloides*, *Turborotalia centralis* and *Borelis* sp. The Middle Miocene carbonates that conformably overlie the early Priabonian, parasequence include a planktonic foraminiferal assemblage of *Globigerinoides trilobus*, *Orbulina universa* and *Borelis melo*. Elsewhere, in the harbour area, the preserved Eocene limestones are also overlain by conformable Miocene carbonate parasequences of Langhian–Serravallian age. Younger argillaceous limestone beds of the Mio/Pliocene age occur in the eastern central part of the harbour area and enclose an assemblage of *Truncorotalia crassaformis*, *Globorotalia inflata* and *Orbulina universa*. The three markers of old and recently raised structural blocks in the harbour area are a Lutetian/Bartonian marine terrace in the south west corner, a lower Pliocene shoreline carbonate section in the north east side and a Holocene raised beach of marine conglomerates in the north east corner of the area. The locations of these paleo-shorelines, less than 2 km apart, indicate a progressive platform narrowing of North Beirut since the Paleogene. This study underpins the geological complexity of the region and contributes to understanding the underlying geology, which will be needed for future regional archaeological, hydrocarbon and hydrogeological exploration.

## Introduction

Lebanon abuts an active transform plate boundary, and its geology reveals a correspondingly complex tectonic history. The lithology of Lebanon is dominated by carbonate rocks, which often can only be differentiated and dated by detailed mircopaleontological investigations. Most recently, there has been increased interest in the geology of on- and off-shore Lebanon, as the hydrogeological, and oil and gas potential of the region is being defined. Furthermore, the 1990s and 2000s saw the revival of archaeological excavations of the major coastal cities of antiquity of Lebanon, namely, Byblos, Beirut, Sidon and Tyre. In Beirut, many reconstructions and infrastructural renewal projects in the harbour and downtown areas have been excavated before construction got underway. These works have given a unique opportunity to sample, and study the newly uncovered geological sections of bedrock, and to expand our understanding of the Cenozoic geology of maritime Lebanon [1]. In this study, the newly revealed excavated sites of the Beirut harbour area tie the occurrence of the Paleogene and Neogene rock formations to the differentially preserved coastal ribbon of Cenozoic outcrops. They identify the differential gradual rise of the Beirut hillsides before and after the earliest uplift of regionally defining Mount Lebanon.

**Figure 1:**
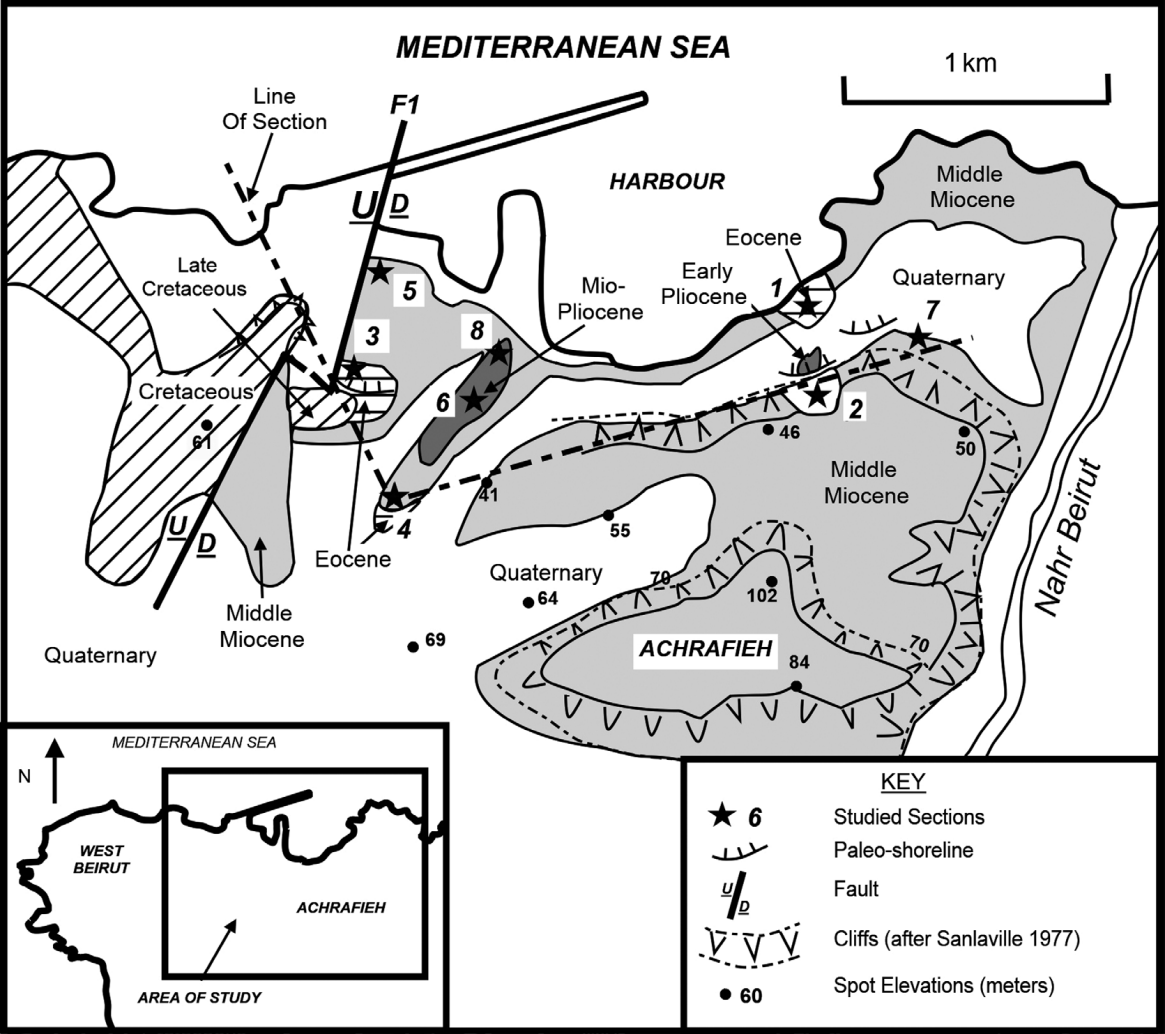
Area of study and location sites based on the revised geological map of Dubertret [2], and the geomorphological map of Sanlaville [3].

The shoreline of North Beirut is a discontinuous marine terrace of 5–20 m in elevation that landward borders an irregular relief from west to east: a Cenomanian hillside of West Beirut, an Eocene-Mio/Pliocene low-lying area of Beirut harbour (Thalweg), and an Eocene-Holocene Achrafieh hillside in East Beirut (see Fig. 1).

The southern outcrops that border the area of study and West Beirut hillside are Quaternary sand dunes and stream gravel deposits. Their occurrence suggests a southern Quaternary shoreline to the Beirut elevated block (see [3, 4]).

The initial 1/50,000 geological map of North Beirut [2] shows two rock formations on either sides of a major NNE SSW (F1) fault, with Cenomanian carbonates to the west of the displacement and outside the area of study and Miocene carbonates to the east of the fault and within the area of study. The current map review based upon the sedimentology and age dating of the excavations’ bedrock and the surrounding outcrops draws the Miocene and Eocene carbonate exposures of the area (Excavation Sites 1, 2 and 3 in Fig. 1). It also locates the previously recorded remnant Maastrichtian carbonate section close to, and east of the F1 fault [1], suggesting that a more complete Cretaceous section had initially covered West Beirut before its displacement along the fault and ensuing erosion (see Fig. 1). Furthermore, it locates the Lutetian/Bartonian marine terrace by the excavations of Site 3, and the Mio/Pliocene argillaceous carbonates by the excavations of Site 6, and the early Pliocene shoreline carbonate section by Site 2 (see location sites in Fig. 1). Equally, it locates the reported Eocene bedrock from an excavation to the south of the area of study and the Holocene marine conglomerates by the north-eastern train station of Beirut harbour area (see Sites 4, 7 in Fig. 1). In essence, the Miocene, Eocene and Maastrichtian limestone rock formations are the bedrock upon which the harbour area is built. The softer argillaceous limestone of the Mio/Pliocene and freshwater/marine Quaternary siliciclastics, which occur in pockets of the hillsides and their surrounds, are the easily eroded materials for natural/man made harbours, such as the Roman harbour in the north-western corner of the area [5, 6] and the waterway parallel to the eastern hillside of the Beirut harbour area, namely “Nahr Beirut”.

The aim of this study was to determine the geological and structural history of the harbour area of Beirut. The biostratigraphy and reconstruction of the carbonate depositional environments are based on the interpretation of the foraminiferal assemblages in the thin sections and the depositional textures, which in turn are key to understanding the hydrocarbon and hydrogeological potential of the region. In our definitions of stratigraphic ranges, the planktonic foraminiferal zonal scheme of BouDagher-Fadel [7] is used. This scheme is tied to the time scale of Gradstein et al. [8] and the revision by Cohen et al. [9].

## The Eocene platform

**Figure 2:**
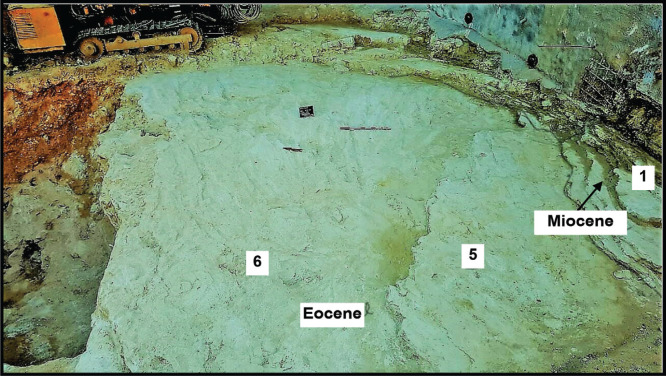
Field photograph of Site 2. In the upper right hand corner, from right to left: the wall covering the Miocene beds under the habitation of “Achrafieh” northern hillside; sampled layer 1 is the base of the Miocene section; sampled layer 5 of thin bedded Eocene carbonates includes the fish deposits of *B. filamentosus*; to the left of the field of view the excavation of a sloped mound uncovering the bedrock of a shoreline carbonate section against the raised cliff side of the Tertiary carbonate section of “Achrafieh”.

**Figure 3:**
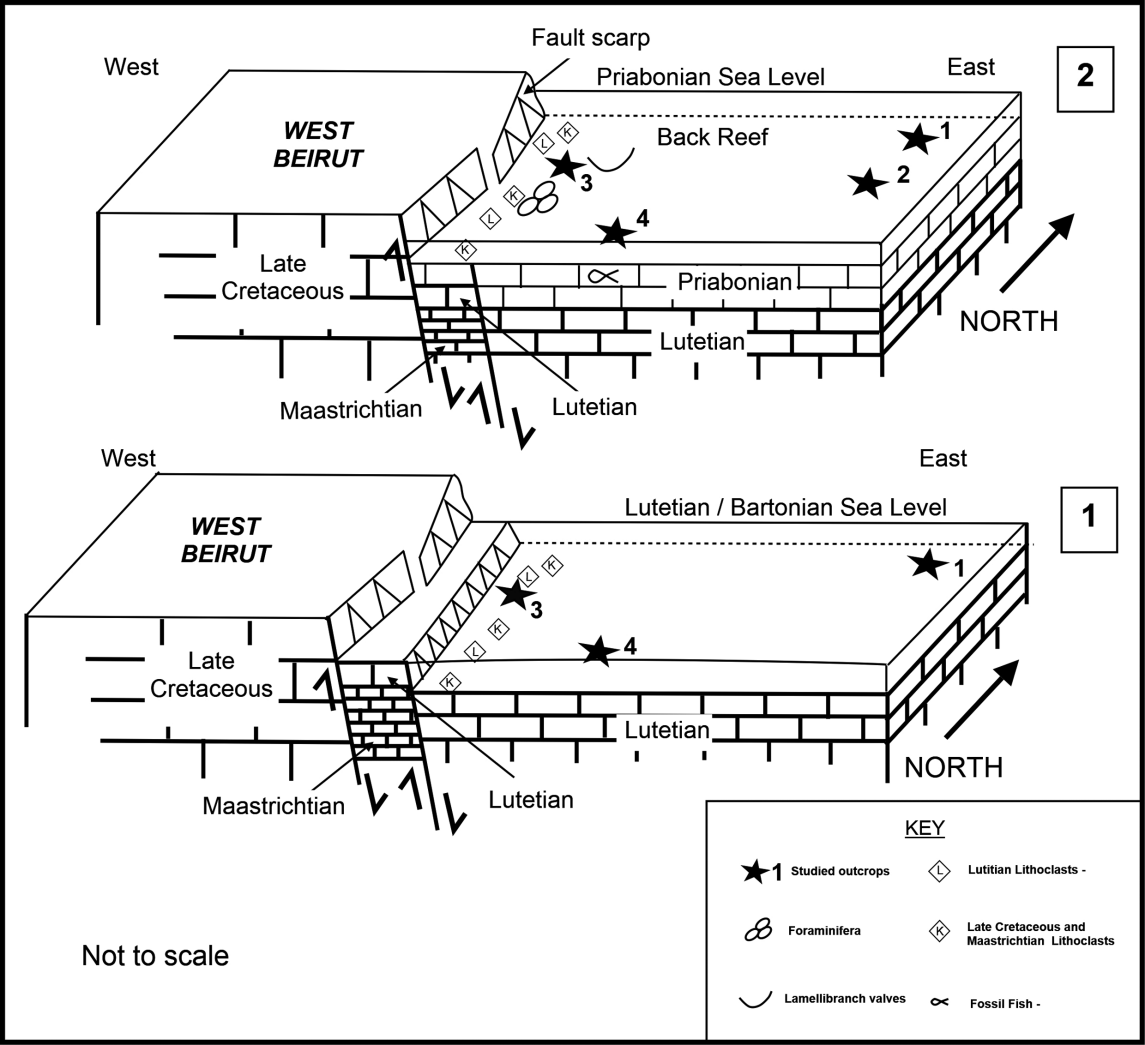
Diagram (1) shows an old elevated Lutetian/Bartonian NS composite faulted western block and the Lutetian/Bartonian shoreline by its eastern side. Diagram (2) shows the inner platform shallow water sediments during the early Priabonian time in the backreef waters of the western structural block.

**Plate 1:**
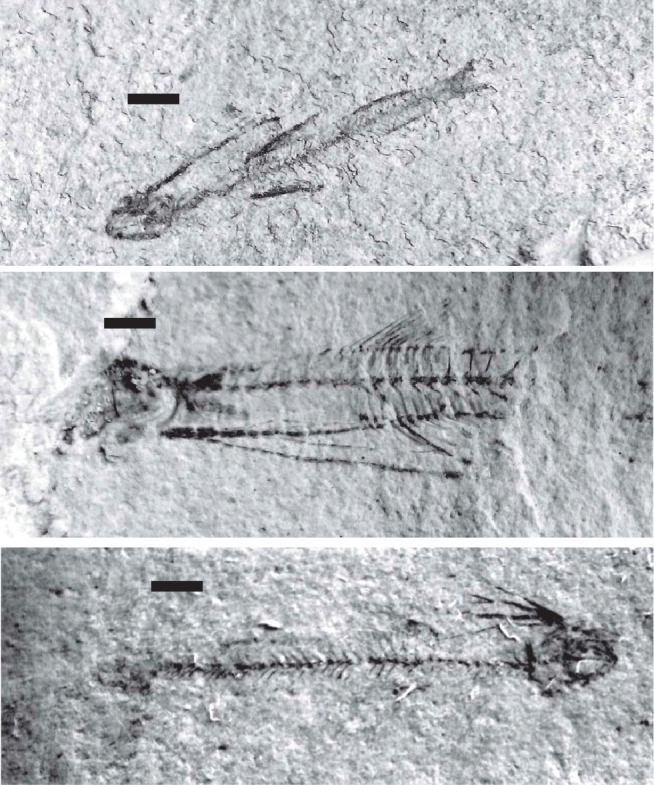
Scale bars: 5 mm. Thin sections of *Bregmaceros filamentosus* (Priem).

The previous works of BouDagher-Fadel and Noujaim Clark [1], on Beirut had recorded carbonate sections of conformable Eocene and Miocene carbonates gently dipping to the south west (Site 1, Fig. 1). In this study, we record conformable Eocene and Miocene carbonates to the south of Site 1 that are dipping to the northeast by about 10 degrees (Site 2, Fig. 1). The Eocene carbonates of Site 1 of medium to thin beds are late Lutetian in age (see table 2 in [1]). However, the newly studied Eocene parasequence of Site 2 exposes 70 cm of thin-bedded carbonates that are early Priabonian in age. This thin carbonate strata includes a fossiliferous-rich interval containing small <5 cm fossil fish of the Bregmacerotidae family (M. Gayet, personal communication, see Figs 2–3, and Plate 1).

### Biostratigraphy and sedimentology

Individual beds of the Eocene carbonate suite are characterised by low angle cross-laminations of bioclastic, siliciclastic, pelletal, lithoclastic packstones and mudstones (see Fig. 2, Samples 6–2 from base to top). The constituents of the grain supported laminations include in a descending order of importance: angular fragments of bioclasts, coral and bryozoan fragments, larger benthic foraminifera, sub-angular to sub-rounded quartz grains >60 μm in diameter, sub-rounded Lutetian and uppermost Cretaceous lithoclasts, red algal fragments, echinoderm debris, lamellibranches debris and planktonic foraminifera. The constituents of the mud-supported laminations include in a descending order of importance: mud sized debris, planktonic foraminifera and rare sub-rounded uppermost Cretaceous lithoclasts. Overall, the environment of deposition of the thin cross-laminated heterogeneous grain supported facies and homogeneous mud supported facies points to shallow water conditions in the back reef–lagoon area close to wave base that is periodically subjected to flooding episodes (see Fig. 3). The high-energy fabrics of their thin beds <15 cm thick suggest a minimal accommodation space rather than slow sedimentation rates. The source of the Cretaceous lithoclasts is likely from nearby exposures and the raised western Beirut block but the provenance of the quartz sands remains problematic. The grainstone facies of Layer 5 of the Eocene suite is of shallow water deposit close to the wave base but it includes an exceptional deposit of small fossil fish of the Bregmacerotidae family (Plate 1).

### Foraminifera assemblages and fossil fish deposits

**Plate 2:**
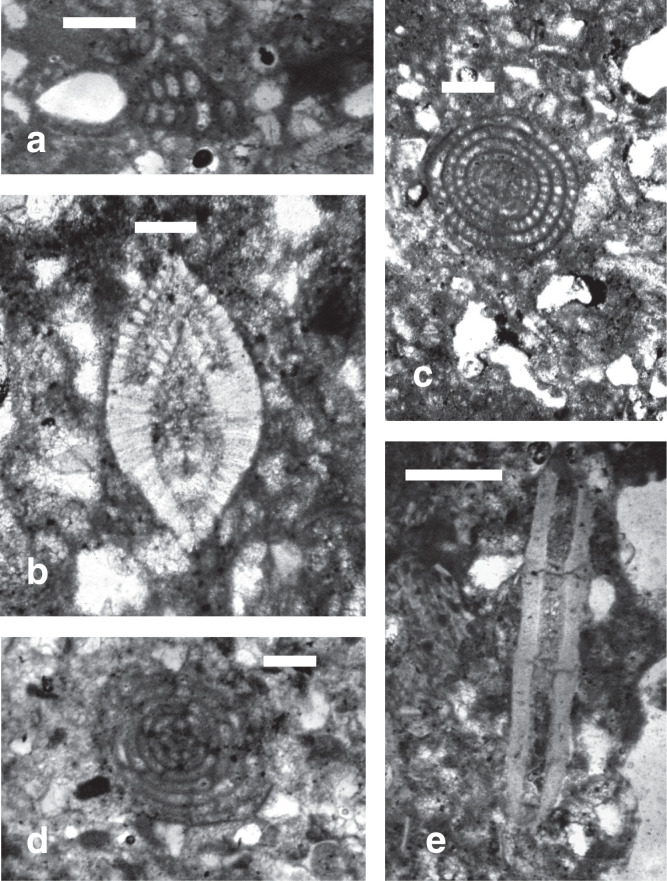
Scale bars: a = 0.3 mm; b–e = 0.5 mm. a. *Textularia* sp., G1. b. *Elphidium* sp., sample G1. c,d.* Borelis* sp., G2. e. Fragment of *Operculina* sp., sample G2.

**Plate 3:**
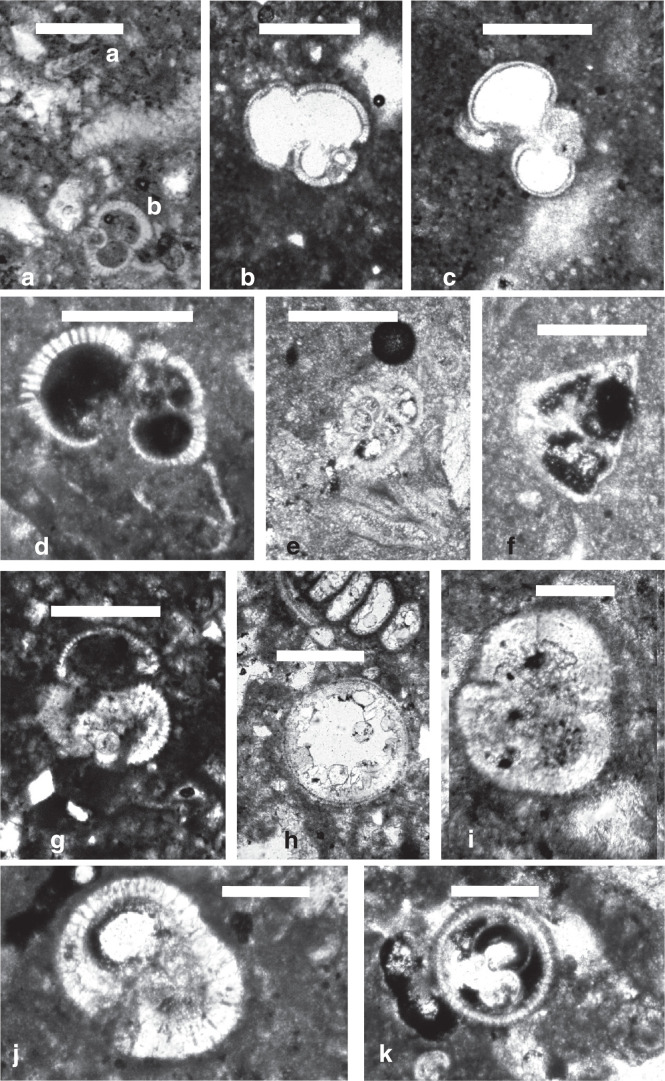
Scale bars: a–k = 0.5 mm. a. a) *Bolivina* sp., b) *Acarinina* sp., sample G2. b. *Globigerinatheka barri* Brönnimann, sample G3. c. *Globigerina praebulloides* Blow, sample G3. d. *Turborotalia centralis* (Cushman and Bermudez), sample G5. e. *Acarinina nitida* (Martin), sample RML5. f.* Morozovella* sp., RML5. g. *Globigerinoides trilobus* (Reuss), sample RML1. h, k. *Orbulina universa* d’Orbigny, 8) RML1; 11) RML9. i.* Sphaeroidinella dehiscens* (Parker and Jones), RML9. j. *Sphaeroidinellopsis subdehiscens* (Blow), RML9.

The foraminiferal assemblages observed in the studied thin sections include small benthic foraminifera such as *Textularia* spp. (Plate 2, a), *Bolivina* sp. (Plate 3, a), and rare benthic foraminifera that consist of long biostratigraphically long-ranging Cenozoic forms, such as *Elphidium* sp. (Plate 2, b), *Victoriella* sp., and fragments of *Operculina* sp. (Plate 2, c). The >10% abundance of planktonic foraminifera from many studied samples narrows their age of deposition. For example, the presence of *Porticulasphaera tropicalis* Blow and Banner, *Globigerinatheka barri* Brönnimann (Plate 3, b), *Dentoglobigerina venezuelana* (Hedberg), *Globigerina praebulloides* Blow (Plate 3, c) and *Turborotalia centralis* (Cushman and Bermudez) (Plate 3, d) nominally indicate a late Bartonian–early Priabonian (Planktonic Zone [PZ] P14–P15, 39.2–35.1 Ma, see [7, 10]). However, the association of the larger benthic foraminifera *Borelis* sp. (Plate 2, c, d) with the planktonic assemblage narrows the age dating of the Eocene carbonate suite to the early Priabonian age (see [7, 11]).

**Plate 4:**
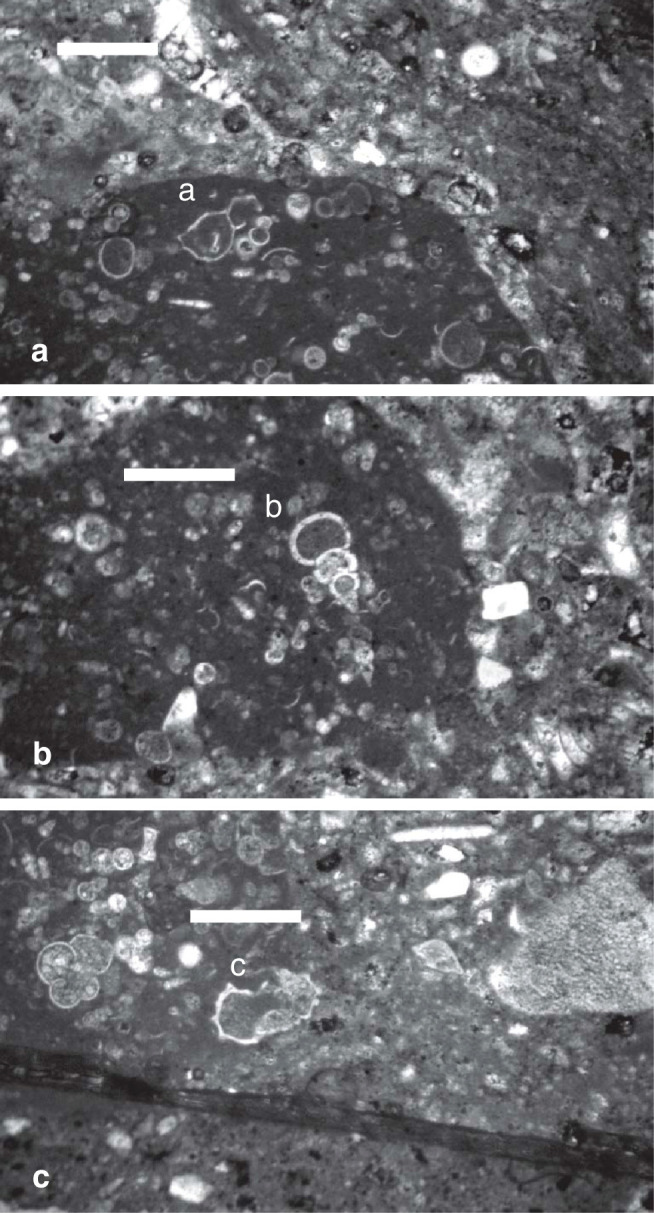
Scale bars: a–c = 0.5 mm. a–c. Micritic wackestone of reworked Cretaceous planktonic foraminifera, sample G1: a) *G. gansseri* (Bolli); b) *P. elegans* (Rzehak); c)* G. aegyptiaca* Nakkady.

Other than these *in situ* assemblages, reworked and allochthonous assemblages include reworked Paleocene and Early Eocene planktonic forms such as *Acarinina nitida* (Martin), (Plate 3, e) and *Morozovella* sp. (Plate 3, f). The allochthonous forms in the Cretaceous lithoclasts contain Late Cretaceous planktonic foraminifera assemblages which include *Globigerinelloides* spp., *Gansserina gansseri* (Bolli) (Plate 4, a), *Pseudotextularia elegans* (Rzehak) (Plate 4, b), *Hedbergella* spp., *Heterohelix globulosa* (Ehrenberg), *Globotruncana* spp., and the frequently present *Globotruncana aegyptiaca* Nakkady (Plate 4, c). These assemblages indicate an age of late Campanian to Maastrichtian (PZ Campanian 3–Maastrichtian 3, see [7, 11]).

The fish deposits of the high-energy bed (layer RML5) belong to the Bregmacerotidae family and are represented by the single genus *Bregmaceros*. They are small-sized, pelagic, 1–2 cm cod-like fish (*Gadiformes*), and their age range is from the Eocene to the Holocene [12]. The species is herein identified as *B. filamentosus* (Priem, 1908), and ranges from the Middle Eocene to Early Oligocene (PZ P10a–P21a, see [7], and has been reported from the Mediterranean region and its surrounding areas including Georgia (Middle to Late Eocene) and Iran (Middle Eocene to Early Oligocene) [12–14].

The bed thicknesses and sedimentology of the Eocene sections of Site 1 and Site 2 indicate a decrease in the accommodation space for carbonates from north to south, and from the late Lutetian to the early Priabonian. However, they are not the only occurrence of the North Beirut Eocene inner platform carbonates. Indeed, BouDagher-Fadel and Noujaim Clark [1] had recorded a marine terrace of Lutetian/Bartonian age in the southwest corner of the studied area (Site 3). This site represents the terrace of the archaeological excavation adjacent to St. George’s Cathedral (Fig. 1). The terrace occurs against the thin hard beds of the basin Maastrichtian basin carbonates, and includes reworked cyclically sorted, coarse cobble to pebble sized Campanian, Maastrichtian and Lutetian lithoclasts bound by Lutetian–Bartonian marine cements. This suggest the concurrent presence of a nearby source of uppermost Cretaceous and Lutetian exposures [1]. The initial reporting of the dates of these outcrops by Dubertret [2] was Eocene and Cretaceous (Senonian) that has since been refined in BouDagher-Fadel and Noujaim Clark [1].

The 1951 “Notice Explicative of Beirut Geological map 1/50,000”, by Dubertret, records another location of Eocene carbonates from an excavation performed south of the central harbour area (Site 4, Fig. 1). This exposure contains thin beds of marls and carbonates rich in reworked terminal Cretaceous planktonic foraminifera, and *in situ* Early Eocene foraminifera. Although the precise dating of these exposures cannot be inferred, these rocks are probably Early Eocene in age, representing the coastal onlap as far south of downtown Beirut.

## The Neogene platform

**Figure 4:**
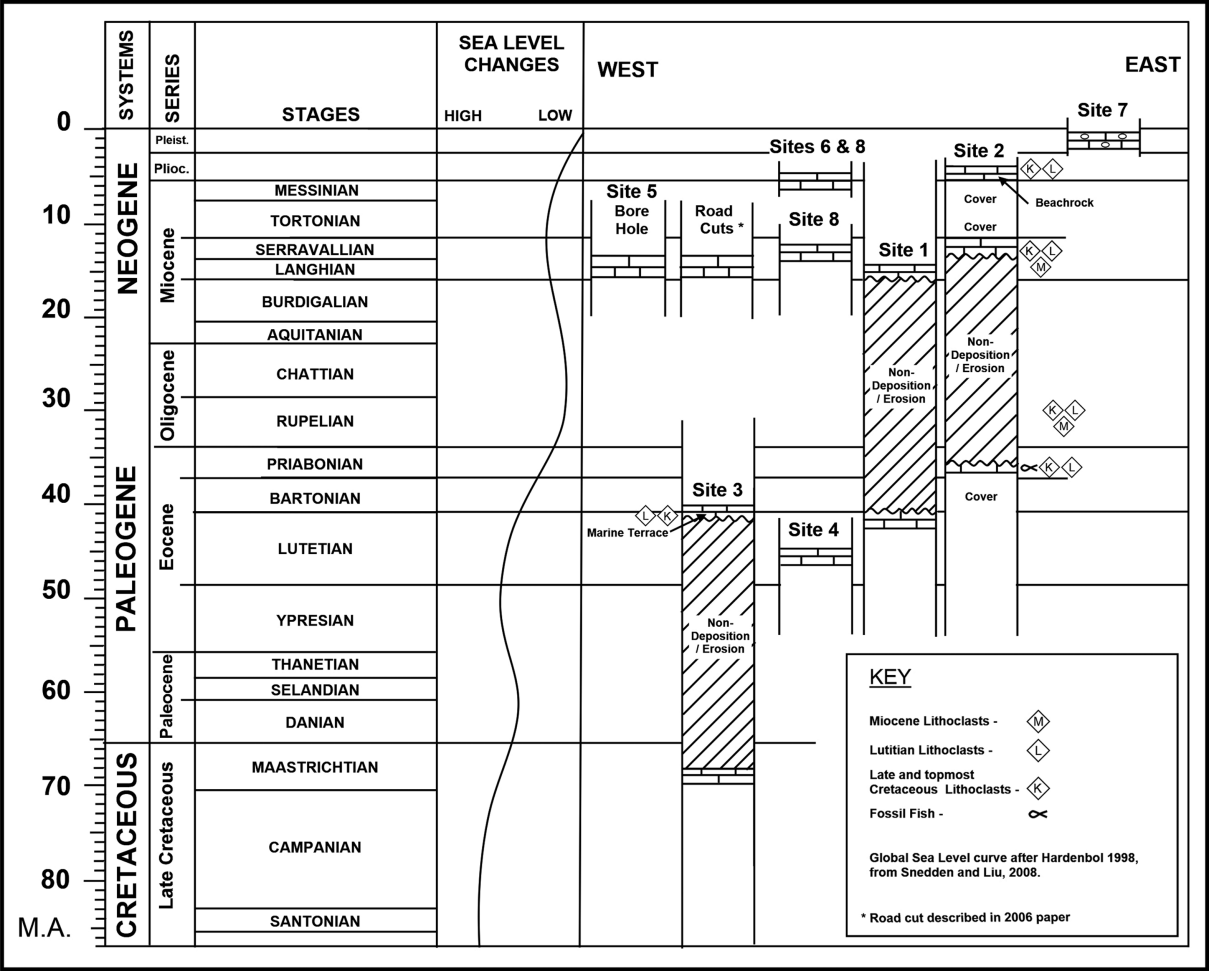
Preserved lithologies of the Eocene and Miocene carbonates of the Beirut harbour area; note occurrences of allochthonous lithoclasts from older reliefs. Global sea level curve after Snedden and Liu [15].

The Langhian–Serravallian carbonates of the North Beirut inner platform covered more or less the same antecedent Eocene platform area in spite of 20 million years of non-deposition and/or erosion between the two time periods. Their conformable parasequences above the middle and upper Eocene carbonates are described from the eastern side of the harbour area. Lower Langhian carbonates are recorded in the north of the studied area from Site1 and Site 5 at the base of the Roman harbour, but their upper Langhian and Langhian–Serravallian carbonates are recorded in the central area of study and southeast of the Beirut harbour at Site 2 (Figs 1, 4; see also [1]). The upper Langhian outcrops in the road cuts in the central harbour area consist of medium to thick carbonate beds dipping steeply to the north east. Younger Serravallian carbonates occur in sections close to the eastern hillside of Achrafieh [1]. The hillside carbonate sections that had been previously assigned a Helvetian age (Serravallian) by Sanlaville [3], suggest latest Middle Miocene deposition to the east and south sides of the harbour area. The locations of these Miocene carbonates are summarized in Fig. 4.

### Biostratigraphy and sedimentology

**Figure 5:**
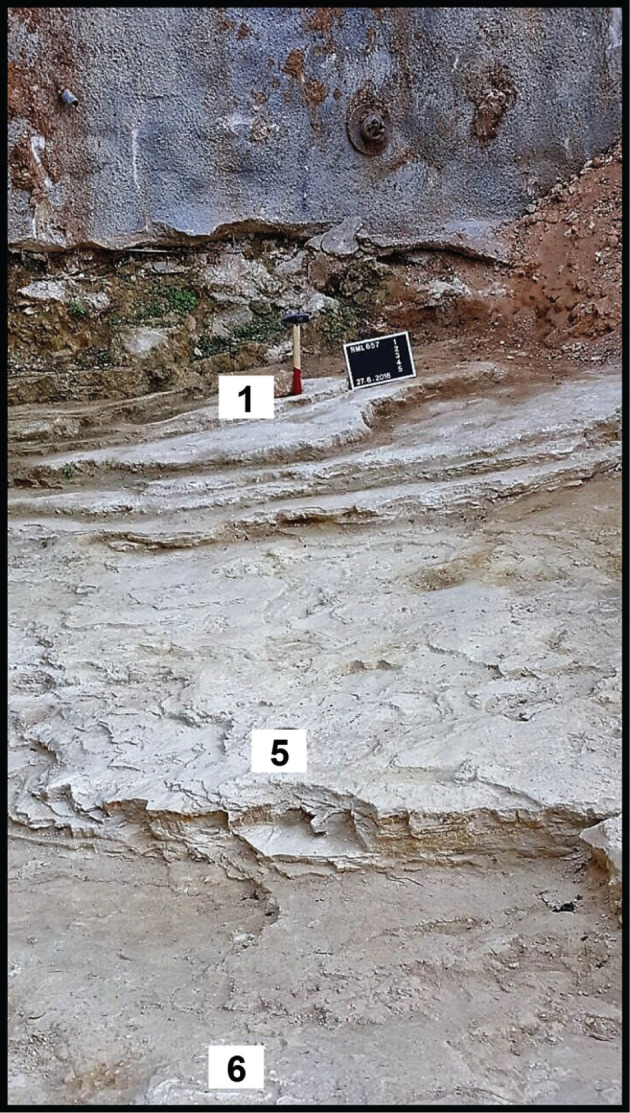
A Miocene brown bed (30 cm) of cross-laminated carbonates resting conformably above thinly cross laminated Priabonian strata of 15–20 cm chalky white carbonates. Base layer 5 in the foreground is the fossil fish layer within the Priabonian parasequence.

The archaeological excavation of the Middle Miocene outcrop of Site 2 lies conformably on the youngest Eocene (early Priabonian) strata. The basal Miocene beds, 50 cm thick, are the only described carbonate beds under a suite of strata concealed by urbanisation (Fig. 5). These beds are characterised by cross-laminated structures, with laminations of coarse packstones and finer wackestones that include reworked uppermost Cretaceous and Middle Eocene lithoclasts suggesting nearby sediment provenance (Figs 1, 4–5). The packstone and wackestone fabrics enclose, in descending order of importance, lamellibranches bioclasts, coral and bryozoan debris, echinoderm fragments, red algal debris, and planktonic and larger benthic foraminifera. The *in situ* foraminiferal assemblage includes *Globigerinoides trilobus* (Reuss)*, Orbulina universa* d’Orbigny (Plate 3, g–h), and* Borelis melo* (Fichtel and Moll), which characterise an age range of Middle Langhian to Serravallian [7, 10]. *Borelis melo* ranges in the Mediterranean from the Middle to Late Miocene (Langhian–Messinian) including the PZ N8b–N17 [16], however, the presence of *O. universa* indicates an assemblage not older than N9. This assemblage, therefore, indicates that these deposits can be correlated with the PZ N9–N17 (15–5.8 Ma). The dominance of Miliolidae and Textularidae in the larger benthic foraminifera assemblage indicates that a shallow neritic environment prevailed during the deposition of these beds. The heterogeneous, fine to >80 μm bioclastic content, the allochthonous rare sub-rounded to sub-angular quartz grains, and lithoclasts of older sediments suggest that sedimentation took place close to the wave base in an inner shelf lagoon.

### The Late Miocene/Pliocene platform remnants

**Figure 6:**
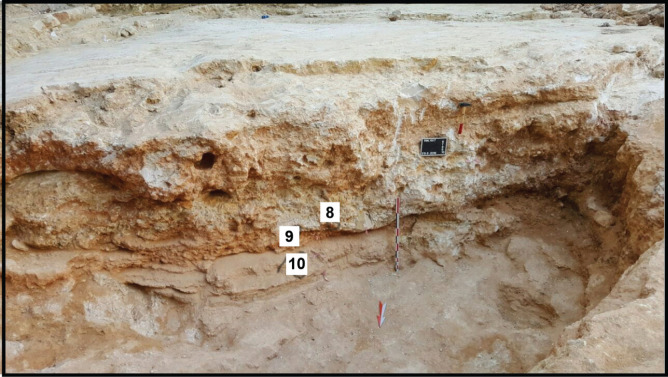
Site 2 excavated bedrock that shows the two stages shore structure, from base: layer 10 of backshore sediments; layer 9 of beach-rock sediments; subangular cobble sized lithoclasts 8 at base of layer 9. The backdrop of field of view is the Paleogene–Neogene outcrop.

The preserved platform deposits of the Mio/Pliocene age occur in the east central harbour area at Sites 6 and 8, where recent excavations have uncovered dark grey argillaceous carbonates of inner platform, lagoonal sediments (Figs 1, 4). These deposits (Site 6) include reworked Cretaceous planktonic foraminifera, and an *in situ* planktonic foraminifera assemblage not older than Late Miocene, PZ N17 (8.6 Ma). However, the presence of *S. subdehiscens* (Blow), *Truncorotalia crassaformis* (Galloway and Wissler), *Globorotalia inflata* (d’Orbigny) and *Orbulina universa* (d’Orbigny) indicate a Late Miocene to early Pliocene age range (PZ N17 – N19, 8.6 – 3.8 Ma, see [7, 11]). The argillaceous carbonates’ occurrences suggest a marine incursion filling the topographic low between the two east and west uplifts of North Beirut (Achrafieh and West Beirut) (see location map Figs 1, 4). To the east of these inner platform sediments, a shoreline carbonate section (Site 2) forms an apron of sediments against the rising northern foothill of Achrafieh. The uncovered bedrock of the Site 2 excavation exposes a shoreline sequence of shore to backshore layers at the base and a beach-rock sedimentary structure on top (Figs 2, 6).

### Lower Pliocene shoreline carbonate section

**Figure 7: (a, b):**
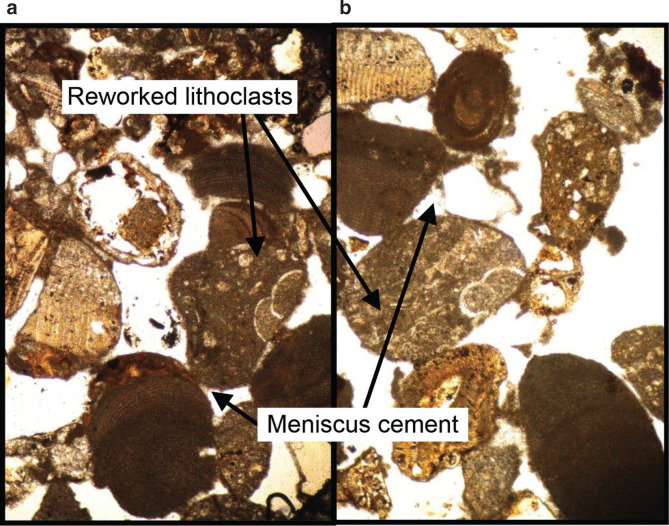
Photomicrographs of back-shore reworked heterogeneous lithoclasts. The grain contacts are cemented with meniscus cement indicative of precipitation in the fresh water flushed vadose zone.

The pit floor of the excavation consists of coarse siliciclastics and carbonate bioclastic debris overlain by a grain supported limestone layer (30 cm thick) composed of low-angle, cross-laminated fabrics of well-rounded sand sized lithoclastic grainstone. The lithoclast grains are bound with meniscus cements that are indicative of meteoric fresh waters, percolating through the vadose zone, in the backshore environment of deposition (see Layer 10 in Figs 6, 7a, b).

**Figure 8:**
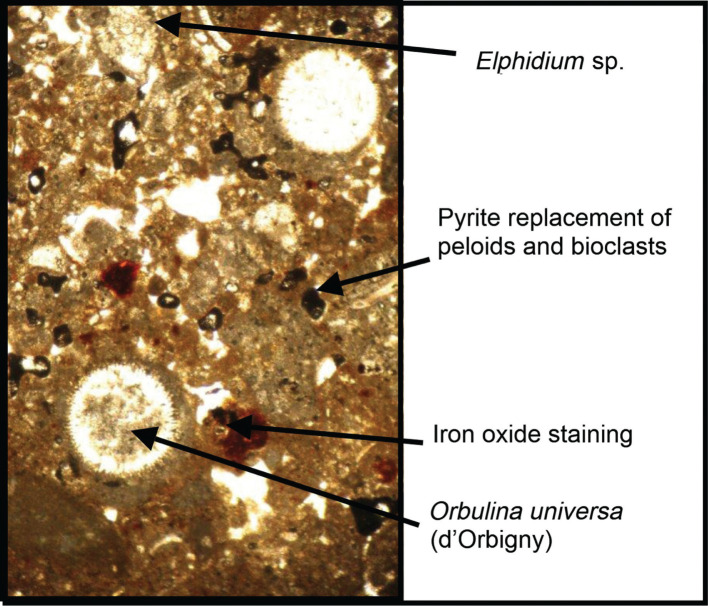
RML9 (11) of layer 9, photomicrograph of intergranular micrite and microspar cement binding the reworked bioclasts of Eocene into the Serravallian–early Pliocene deposits. Note, selective pyrite replacement of allochthonous peloids and bioclasts.

The uppermost layer of the bedrock (Layer 9) is 1 m+ thick. It is characterised by saucer shaped sedimentary structures and low angle cross-stratified bedding, that is interpreted as a lithified beach-rock structure during a sea level rise (Fig. 6). Its heterogeneous fabric is made of coarse cobble to sand sized clasts in a matrix of bioclastic lithoclastic grainstone (see lithoclast 8 in Fig. 6). The binding cement of its bioclastic and lithoclastic components is of micrite and microspar, indicative of the mixing marine phreatic and freshwater zones (see Fig. 8). Two populations of autochthonous and allochthonous bioclasts include, in a descending order of importance: planktonic foraminifera such as *Globigerina* spp. (Plate 4, a–c), larger benthic foraminifera, peloids, bioclasts (lamellibranches and bryozoan debris), lithoclasts, algal debris and iron rich reworked lithoclasts. The youngest planktonic foraminifera of the assemblage are *S. dehiscens* (Parker and Jones) (Plate 3, i), *S. subdehiscens* (Blow), (Plate 3, j), and *O. universa* (d’Orbigny), (Plate 3, h, k). The presence of *S. subdehiscens* and *S. dehiscens* indicate an age of Early Pliocene, PZ N19, 5.3–3.8 Ma. The allochthonous population of bioclasts include Cretaceous (late Campanian 3b – Maastrichtian, 74.5–66 Ma). The upper Eocene (Priabonian) and middle Miocene (Langhian) cobble to pebble sized lithoclasts in the matrix of the beach rock suggesting a nearby source of supply.

**Figure 9:**
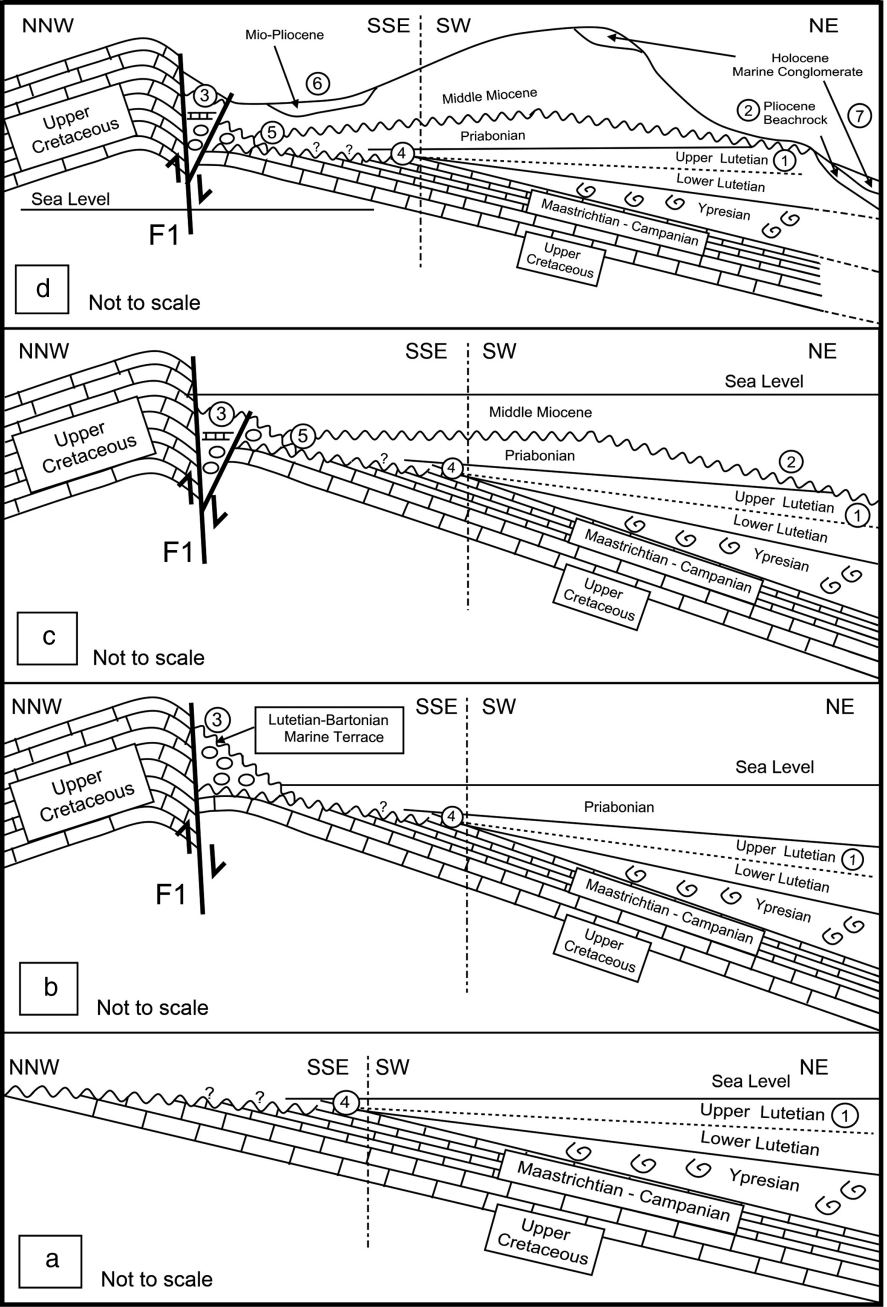
(a) Draws the Lutetian coastal onlap as far south of the harbour area by Site 4; (b) marks the West Beirut displacement post-Lutetian and marine terrace formation during the Lutetian/Bartonian time and the onset of the Priabonian coastal onlap recorded from Site 2; (c) shows the renewed displacement of F1 fault preceding the middle Miocene coastal onlap and the Miocene/Eocene diachronous unconformity in Sites 1 and 2; (d) draws remnant of a coastal onlap in Site 6, the early Pliocene shoreline section of Site 2, and the latest raised beach marine conglomerates of Site 7 and the topmost “Achrafieh” hillside.

The components of the shoreline carbonate section indicate a lower Pliocene shoreline forming in the earlier wave cut erosional stage during a relative sea level drop, or at the onset of the staged uplifts of Achrafieh’s hillside. This was followed by a relative sea level rise cementing the contents in a beach-rock structure (Figs 4, 9d).

### Holocene shoreline sediments

Holocene marine conglomerates and siliciclastics of a raised beach at the site of the Beirut Old Train Station (Site 7, Fig. 1) had been described by Zumoffen [17] and Sanlaville [3] where it occurs approximately 100 m downdip of the early Pliocene shoreline carbonate section of Site 2. The relationship of these two shorelines illustrates the multi-phased uplifts of the Achrafieh hillside (see Figs 1 and 9d).

## Structure, regional tectonics and paleogeography

### Structural history

Carbonate rocks exposed in the area of the Beirut Harbour are dated as Paleogene and Neogene based on the microfossil assemblage. This new biostratigraphic data gives a clearer picture of the structural evolution of Beirut. Lithoclasts of the latest Cretaceous, middle Eocene and oldest middle Miocene age detected within these carbonates point to uplift and erosion phases of beds of this age from nearby areas. Four diagrams summarise the structural and geological evolution of the harbour area (Fig. 9).

*In situ* foraminifera assemblages provided a relative age of Eocene and Miocene for the. parasequences. However, the youngest allochthonous lithoclasts in these same beds, provide information on the carbonates present in the area prior to erosion, and also serve to date the earliest uplift that would have occurred in the source area.

A debate has been developed on the timing of the uplift of Mount Lebanon in relation to the structural deformation along the Dead Sea fault zone. For example, Gomez et al. [18] indicated that Mount Lebanon started rising during the Middle Miocene and continued during the Late Miocene. Previous works by BouDagher-Fadel and Noujaim Clark [1] support this timing when recording Langhian lithoclasts in younger Serravallian and Tortonian–Messinian carbonates of coastal Tyre of south Lebanon and at the Jabal Terbol mid-Langhian–Tortonian section of north maritime Lebanon.

### Eocene and Miocene movements

In the case of the Eocene carbonates at Site 3, the inclusion of lithoclasts are dated as Campanian, Maastrichtian and Lutetian in the shoreline/marine terrace sequence. This confirms that the down thrown side of the NNE SSW (F1) fault would have included the latest Cretaceous and middle Eocene beds before their marine erosion during the Lutetian/Bartonian time. The displacement along the F1 fault (Site 3) is inferred to have occurred nearer the end of the Middle Eocene (Bartonian), as indicated by foraminifera. The areal extent of the Lutetian carbonates are inferred to have covered the F1 fault, before the displacement along its side and the uplift of West Beirut and F1 compartments. In addition, younger (Late Eocene) Priabonian beds at Site 2 lateral to Site 3 include Lutetian and Cretaceous lithoclasts confirming the presence of nearby exposures of Lutetian and terminal Cretaceous beds to Site 2 (see Fig. 9b,c).

The Langhian and Serravallian (Mid-Miocene) carbonates of the harbour area include only *in situ* inner platform microfauna. The exception is at Site 2, by the eastern side of the harbour area, where the conformable Langhian–Serravallian bed contains Lutetian and Cretaceous lithoclasts. The nearby source of that foreign supply to the recipient Langhian–Serravallian sediments is likely to have been from the few preserved Lutetian and Bartonian exposures of the harbour area.

The lower Pliocene shoreline by the uplifted Eocene–Miocene conformable section of Site 2 includes middle Miocene lithoclasts. These were sourced either from the rising block of the middle Miocene beds (Achrafieh) and/or from the exposures in the south west of the harbour area. The occurrence of Mio/Pliocene argillaceous carbonates (Site 6 and Site 8) due south of the harbour area implies marine sedimentation filling a platform low between the uplifted reliefs of West Beirut and Achrafieh Eastern hillside (Fig. 9d).

### North Beirut in the context of maritime Lebanon

Differential Cenozoic structural highs occur along the maritime Lebanon because of the rise of the Mount Lebanon during the Paleocene until the Late Miocene [1]. The latest rise was concurrent to the platform narrowing and establishment of shorelines close to the present-day coastline. The structural uplift of West Beirut in the late Middle Eocene was initiated while the African and Arabian plates were still united. The uplift of this area was possibly related to local tectonics. However, its reactivation during the mid-Miocene and early Pliocene took place several millions of years later after the African and Arabian plates had separated.

**Figure 10:**
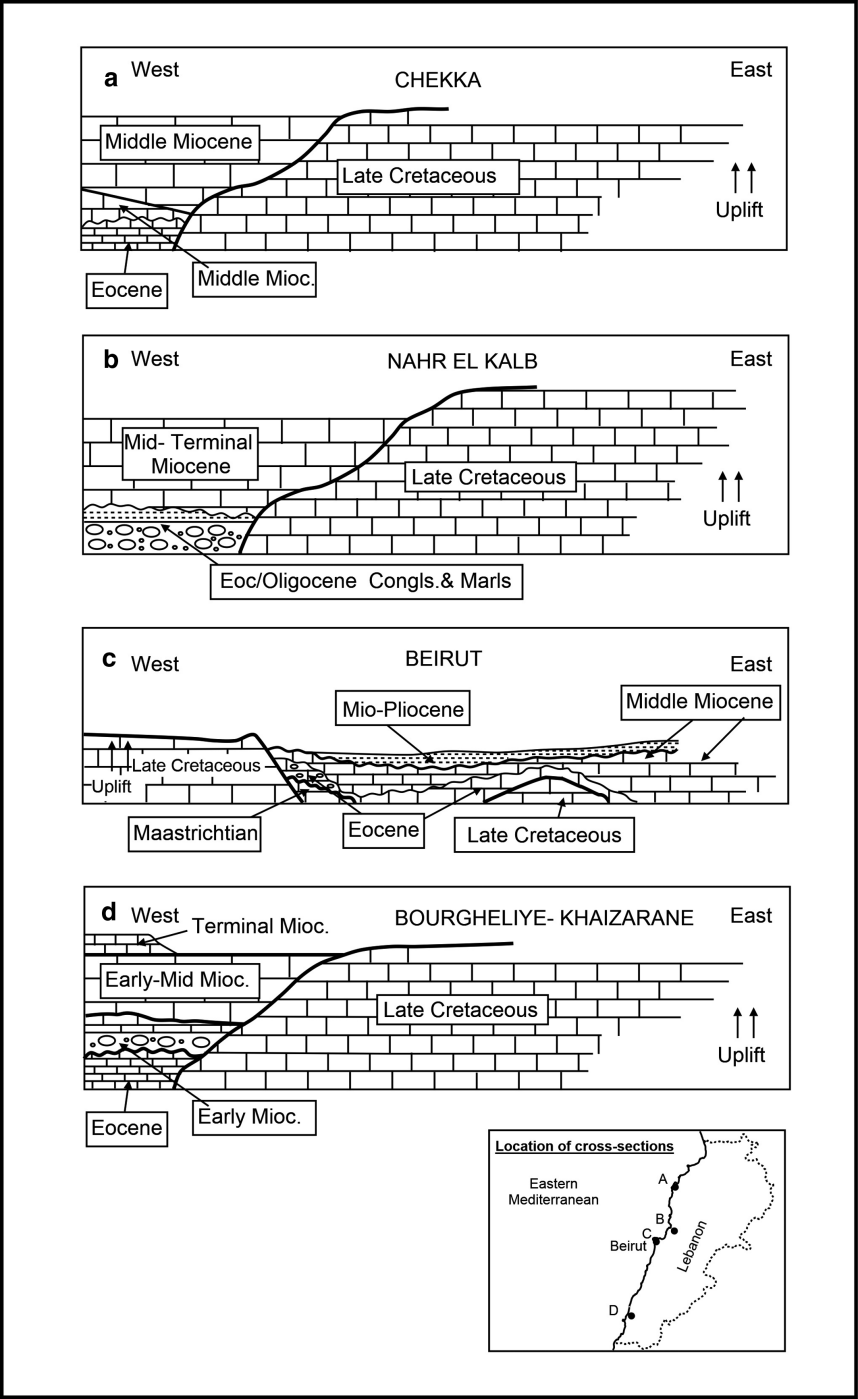
Summary of the preserved Cenozoic marine sections of the Lebanon coastline during the Middle to Late Miocene. Note the exceptional Beirut profile where there was a pre-existing relief in the west controlling the platform from the NE and SW.

The Dead Sea Transform fault in Lebanon, called the Yammouneh Transform fault, separates the two plates. Transpressive and transtensional stresses along the fault zone caused the rise of the Mount Lebanon on the one hand, and the extensional landward basins such as today’s Yammouneh inland rhombic basin in the Bekaa Valley, on the other hand. The structural geology describing these occurrences had listed two phases of uplift of Mount Lebanon during the Middle Miocene and the Late Miocene, but the present study and that of BouDagher-Fadel and Noujaim Clark [1] further calibrates the uplift phases of Mount Lebanon. For example, the dating of individual Middle and Late Miocene beds or parasequences have shown a steady supply of older uppermost Cretaceous, Paleocene, Middle Eocene and lower Middle Miocene lithoclasts into recipient Langhian–Serravallian strata and Tortonian strata in north Lebanon (Jabal Terbol); in Langhian–Serravallian strata and lower Pliocene strata in Beirut (Site 2); in lower Langhian strata from sections of coastal Tyre of south Lebanon. These dating markers from the studied coastal outcrops provide the timing of the differential uplifts that had occurred during the earliest Middle Miocene in the south of Lebanon, the Middle Miocene in Beirut and Jabal Terbol north of Lebanon, the Late Miocene in the Jabal Terbol and latest early Pliocene in Beirut (see Fig. 10).

## Discussion and conclusions

**Figure 11:**
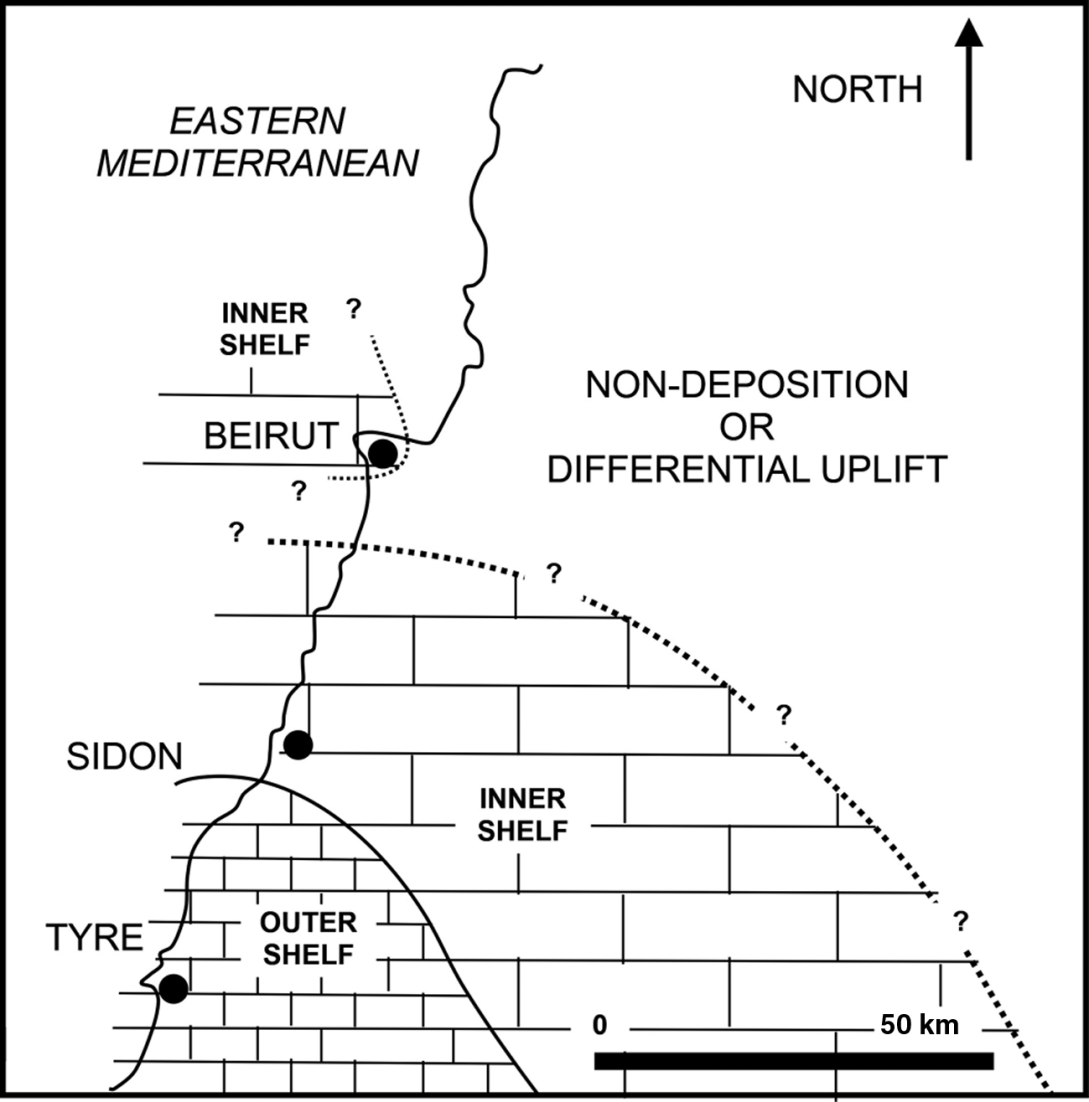
Reviewed Priabonian environment of deposition from BouDagher-Fadel and Noujaim Clark [1] in light of the present-day description of its environment of deposition in the harbour area.

The Cenozoic geology of maritime Lebanon records Paleocene–Eocene (Priabonian) carbonates on a broad marine platform concurrent with differential relief in south coastal Lebanon [1]. In the Beirut harbour area, the earliest coastal onlap of the Middle Eocene would have advanced over a Maastrichtian platform floor and remnants of Ypresian chalks. This is confirmed by the Maastrichtian carbonates at Site 3 and the older reworked Ypresian and latest Cretaceous planktonic foraminifera in younger upper Lutetian beds at Sites 1 and 4, [1, 2, 4]. At Site 2 the younger Priabonian thin beds of the inner platform carbonates occurred in a minimal accommodation space near a shoreline. This occurrence is in contrast to the shelf edge occurrence of their contemporaneous chalk deposits in south coastal Lebanon [1]. As these two deposits are the only evidence of the Late Eocene platform sedimentation along maritime Lebanon, their occurrences in the inner shelf and shelf edge environments follow the paleogeography drawn in BouDagher-Fadel and Noujaim Clark [1] with an interpreted and drawn shoreline around the Beirut harbour area (see Fig. 11).

The earliest Neogene marine incursion of the Beirut harbour area is of early Langhian age. This incursion filled the released space of the downthrown side of the Eocene relief of West Beirut.

In the context of maritime Lebanon, the thin to thick bedding (15–60 cm) of the Middle Miocene in the studied area contrasts with the massive beds (1–1.5 m) of the mid Miocene north of Beirut, in the Nahr el Kalb and Chekka areas, indicating deposition under differentially created accommodation spaces (see Fig. 10). The Beirut sediments were probably deposited on a high whereas in areas A and B of Fig. 10 the contemporaneous sediments piled massively in a subsiding low apace with Mount Lebanon rise (Fig. 10; [1]).

In North Beirut, the two uplift phases of the Neogene are implicitly and/or directly indicated by the studied outcrops: the first occurred before the Langhian/Serravallian when F1 fault was reactivated in the western side of the harbour area to supply uppermost Cretaceous and Eocene lithoclasts to the conformable Miocene bed of Site 2. The second and younger phase, occurred in the early Pliocene time when the West Beirut block was further uplifted and the Achrafieh rise was initiated. The evidence of the later phase of uplift is from the lower Pliocene beachrock of Site 2 that encloses reworked uppermost Cretaceous lithoclasts, Eocene lithoclasts and Middle Miocene (Serravallian) lithoclasts (Fig. 9d).

During the Ypresian, the rise in sea level reached a maximum with hemipelagic sedimentation over most of maritime Lebanon. Reworked Ypresian benthic and planktonic foraminifera in Lutetian carbonates of Sites 1 and 4 record this occurrence in the Beirut harbour area (Fig. 9a). During the Lutetian, the carbonate platform was differentiated into inner and outer depositional zones with carbonate build-ups as recorded south of Beirut and on the eastern and south eastern side of Mount Lebanon. In the case of the Beirut harbour area the Lutetian carbonates are characterised by inner platform deposits (see BouDagher-Fadel and Noujaim Clark [1]) describing Lutetian paleogeography pre-Dead Sea Transform left lateral movements and rotation).

The West Beirut High flanked by the marine terrace of Lutetian/Bartonian age at Site 3 sheltered the younger Priabonian inner platform carbonates of Site 2. The relief separated an inner platform environment of the harbour area from an outer platform environment to the west and north of The West Beirut High (see Fig. 3). Remarkably, the high-energy carbonates of the youngest Eocene inner platform shallow water sediments include whole or broken pelagic fossil fish of the Bregmacerotidae family (see Plate 1). Elsewhere, the youngest Eocene platform limestones left few preserved sections in south maritime Lebanon in addition to the section of Site 2 by the northern foothill of Achrafieh in Beirut (see above and [1]).

Twenty million years of non-deposition and/or erosion separate the preserved Eocene carbonates from the conformable overlying Middle Miocene carbonates of the harbour area. The younger mid Miocene carbonates of Site 2 infer a reactivation and uplift of F1 fault as they contain eroded Campanian, Maastrichtian and Lutetian lithoclasts. The later phase of the early Pliocene uplift affected the Achrafieh area on the one hand, and the reactivation of F1fault and West Beirut area on the other hand. A low area between these highs, was filled with inner platform argillaceous carbonate sediments (Fig. 9d).

In the studied area, the youngest allochthonous lithoclasts in recipient sediments have dated the emerged reliefs that are the source of supply. Along maritime Lebanon, the same principle applied to indicate that Mount Lebanon rose differentially through several phases of uplift: at the beginning of the Middle Miocene time, during the Late Miocene, and during the early Pliocene time before the final Holocene continental emergence.

The biostratigraphy and sedimentology of the studied exposures of the Cenozoic carbonates of the Beirut harbour area described here add new information to the geological history and paleogeography of maritime Lebanon initially described in BouDagher-Fadel and Noujaim Clark [1]. The underpinning of the geological complexity and structural history of this area is a contribution to the developing understanding of the archaeological context, and of the hydrocarbon and hydrogeological potential of the region.

## Data Availability

All data generated or analysed during this study are included in this published article.
